# Ebola Outbreak Response; Experience and Development of Screening Tools for Viral Haemorrhagic Fever (VHF) in a HIV Center of Excellence Near to VHF Epicentres

**DOI:** 10.1371/journal.pone.0100333

**Published:** 2014-07-09

**Authors:** Rosalind Parkes-Ratanshi, Ali Elbireer, Betty Mbambu, Faridah Mayanja, Alex Coutinho, Concepta Merry

**Affiliations:** 1 Infectious Diseases Institute, Makerere College of Health Sciences, Kampala, Uganda; 2 Johns Hopkins University, School of Medicine, Baltimore, Maryland, United States of America; 3 Trinity College Dublin, Dublin, Ireland; 4 Northwestern University, Chicago, Illinois, United States of America; Division of Clinical Research, United States of America

## Abstract

**Introduction:**

There have been 3 outbreaks of viral hemorrhagic fever (VHF) in Uganda in the last 2 years. VHF often starts with non-specific symptoms prior to the onset of haemorrhagic signs. HIV clinics in VHF outbreak countries such as Uganda see large numbers of patients with HIV 1/2 infection presenting with non-specific symptoms every day. Whilst there are good screening tools for general health care facilities expecting VHF suspects, we were unable to find tools for use in HIV or other non-acute clinics.

**Methods:**

We designed tools to help with communication to staff, infection control and screening of HIV patients with non-specific symptoms in a large HIV clinic during the outbreaks in Uganda. We describe our experiences in using these tools in 2 Ebola Virus Disease outbreaks in Uganda.

**Results:**

During the Ebola Virus Disease (EVD) outbreaks, enhanced infection control and communication procedures were implemented within 24 hours of the WHO/Ministry of Health announcement of the outbreaks. During course of these outbreaks the clinic saw 12,544 patients with HIV 1/2 infection, of whom 3,713 attended without an appointment, suggesting new symptoms. Of these 4 were considered at risk of EVD and seen with full infection procedures; 3 were sent home after further investigation. One patient was referred to the National Referral Hospital VHF unit, but discharged on the same day. One additional VHF suspect was identified outside of a VHF outbreak; he was transferred to the National Referral Hospital and placed in isolation within 2 hours of arriving at the HIV clinic.

**Discussion:**

Use of simple screening tools can be helpful in managing large numbers of symptomatic patients attending for routine and non-routine medical care (including HIV care) within a country experiencing a VHF outbreak, and can raise medical staff awareness of VHF outside of the epidemics.

## Introduction

Viral hemorrhagic fevers (VHFs) refer to a group of illnesses that are caused by viruses of diverse families, including Lassa fever, Rift Valley Fever, marburg viruses, and ebola viruses [1], [2]. VHF's characteristically lead to overall vascular system damage and often accompanied by hemorrhage (bleeding). VHF have high case fatality rate (between 25–100% [3]) and spread easily (in an outbreak in Zaire up 25% of cases were health care workers [4]). Providing care to VHF suspects without adequate infection control processes can cause extreme anxiety [5]. Exposure to VHF patients often results in health care workers being quarantined, which can be frightening and unpleasant [6]. VHF outbreaks provoke widespread national and international interest, often with dramatic media reporting [7]. Therefore, health care workers in VHF areas are usually very aware of health professional fatalities in previous outbreaks, through both media reports and peer to peer communication [8], [9]. Therefore providing medical care in any setting near to a VHF outbreak is stressful for health care workers [10]–[12]. Consequently, managing this anxiety and stress in health care workers, whilst continuing to provide a comprehensive service is essential in maintaining an effective health care service during a VHF outbreak.

The initial symptoms and signs of VHF are often non-specific, including fever, rash and vomiting, prior to emergence of a more haemorrhagic pattern [13]–[16]. In Sub-Saharan African countries, where there is a high burden of infectious disease, non-specific symptoms are commonly seen in many patients without VHF attending health care facilities, especially in those presenting with malaria [17], [18] and complications of HIV [19]. This means that during a VHF outbreak there are many people attending health care facilities near to the epicenter who may have similar symptoms to those with VHF, but who are not exposed or infected. In Uganda there are about 438,000 people who regularly access care and treatment for HIV1/2 infection, and many of them attend clinic every month [20]. Therefore, up to 20,000 patients attend for HIV 1/2 related care every day across the country. Missed appointments and especially HIV drug treatment interruptions may adversely affect the health of HIV infected patients. Health centres distant from a VHF epicenter in Angola saw reduced attendance of patients for other causes during the VHF outbreak [21], suggesting that patients also suffer from anxiety during VHF outbreaks and have been found to exhibit altered health seeking behaviours. Ensuring that patients attend for their HIV appointments is vital for good HIV care and treatment, and therefore reassurance to patients during a VHF outbreak is another priority for maintaining good clinical services.

In 2012 Uganda experienced 3 separate outbreaks of viral haemorrhagic fever; two Ebola Virus Disease (EVD) outbreaks [22] and one Marburg Virus Disease (MVD) outbreak. The Adult Infectious Disease clinic (AIDC) at the Infectious Diseases Institute (IDI) in Kampala sees up to 500 patients with HIV 1/2 infection per day. During the recent VHF outbreaks in Uganda it was essential to protect and reassure both the health care workers and patients as much as possible, whilst maintaining routine clinical services for all patients. However, whilst good tools were available for VHF treatment centres [1], [23], we were unable to find any readily available tools or literature that would assist us to deal with a large and often symptomatic HIV clinic population during a VHF outbreak. Therefore, the IDI team developed 3 tools; an action plan for staff and management, a screening tool for symptomatic patients, and a laboratory information leaflet to assist guide and assist health care workers to continue routine clinic activities as safely as possible. In this article we present these tools and our experience with using them.

## Methods

**Figure 1:**
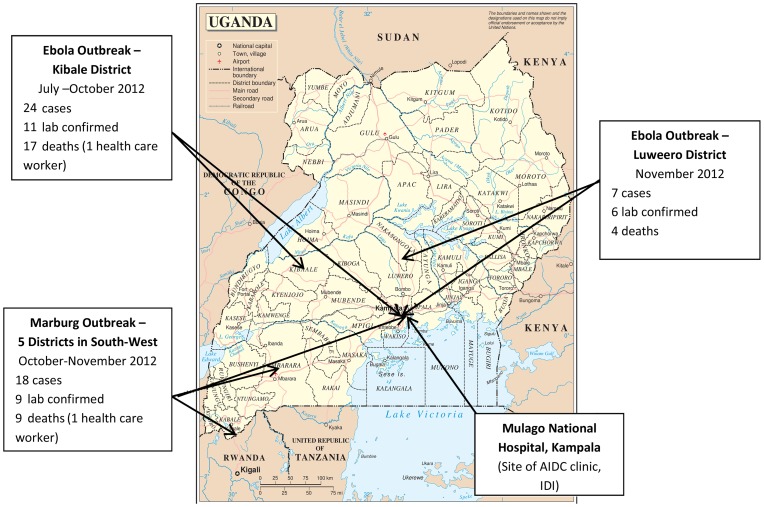
Map of VHF outbreaks in Uganda July 2012–June 2013.

The Adult Infectious Disease Clinic (AIDC) at the Infectious Diseases Institute is located within the Mulago National Referral Hospital Complex, Kampala. Between July –September 2012 there were 9925 patients infected with HIV 1/2 registered at the clinic and there were an average of 465 patients seen per day. Patients are seen on appointment (scheduled) or if they are unwell (unscheduled). Between 29th July and 4^th^ October 2012 in Kibale District Western Uganda and 17^th^ November and 30^th^ November 2012 in Luweero District, Central Uganda, there were 2 outbreaks of EVD virus. Between 22^nd^ October and 23^rd^ November 2012 there was a MVD outbreak in South West Uganda. The AIDC clinic is located approximately 206 km, 64 km and 404 km miles respectively from these 3 VHF outbreaks (figure 1). 41% of the AIDC patients live outside of Kampala, and many travel from across the country for their HIV care in the AIDC clinic. During each VHF outbreak both suspected and confirmed VHF patients travelled to Kampala and sought care at the National Referral Hospital and from other health care providers. Therefore, there was a credible risk of VHF suspects attending for care at the AIDC.

**Figure 2:**
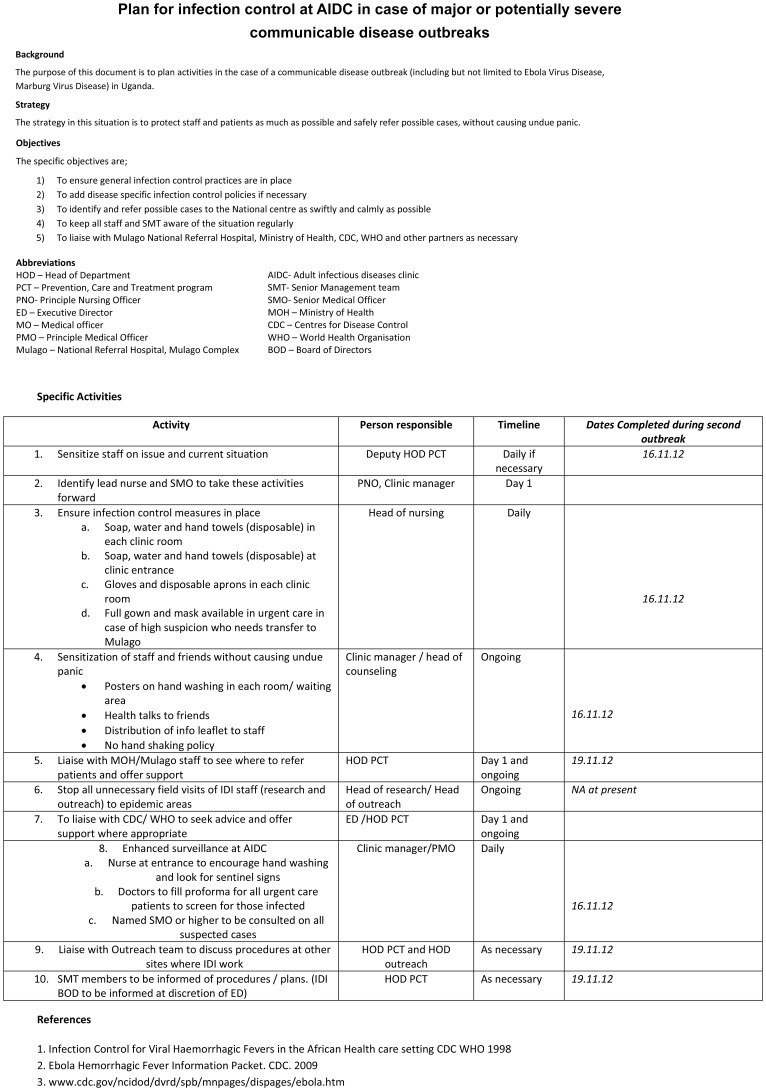
Plan for infection control at AIDC in case of major or potentially severe communicable disease outbreaks.

As details of the first outbreak emerged, the clinic management team looked for tools which we could use for information and patient screening in our clinic. The CDC/WHO Infection Control document for VHF in African settings was used as a reference, but this was not specific for an HIV out-patient setting [23]. Therefore we produced and distributed our own tools on 30^th^ July 2012. The first tool (figure 2) was an action plan developed to establish the process of our response to the epidemic, and was circulated to all staff and IDI senior management, along with the CDC Ebola leaflet for background information[24]. As per the action plan, we met to discuss with situation with all staff, and to give them concise and scientific information on the virus.

During each outbreak we identified an infection control nurse to be stationed at the clinic entrance. Her role was to perform triage of patients and to encourage standard infection control procedures such as hand-washing. She was responsible for ensuring that there were adequate hand washing facilities and supplies. All staff and patients were requested to wash their hands before and after entering the clinic. At entrances where soap and water were not available, alcohol gel was used. All staff were reminded to wash hands or use alcohol gel in between patients. The infection control nurse also gave twice daily health talks to patients in the waiting area, and answered questions related to VHF. This nurse also performed a basic triage of unwell patients with respect to haemorrhagic symptoms, and discussed all potential cases with a senior staff member before they were allowed to enter the main clinical unit. Any VHF suspects were directly referred to the National Referral Hospital Ebola unit and not admitted to the AIDC clinic. These activities were continued until Ministry of Health announcements stating the end of the outbreak.

**Figure 3:**
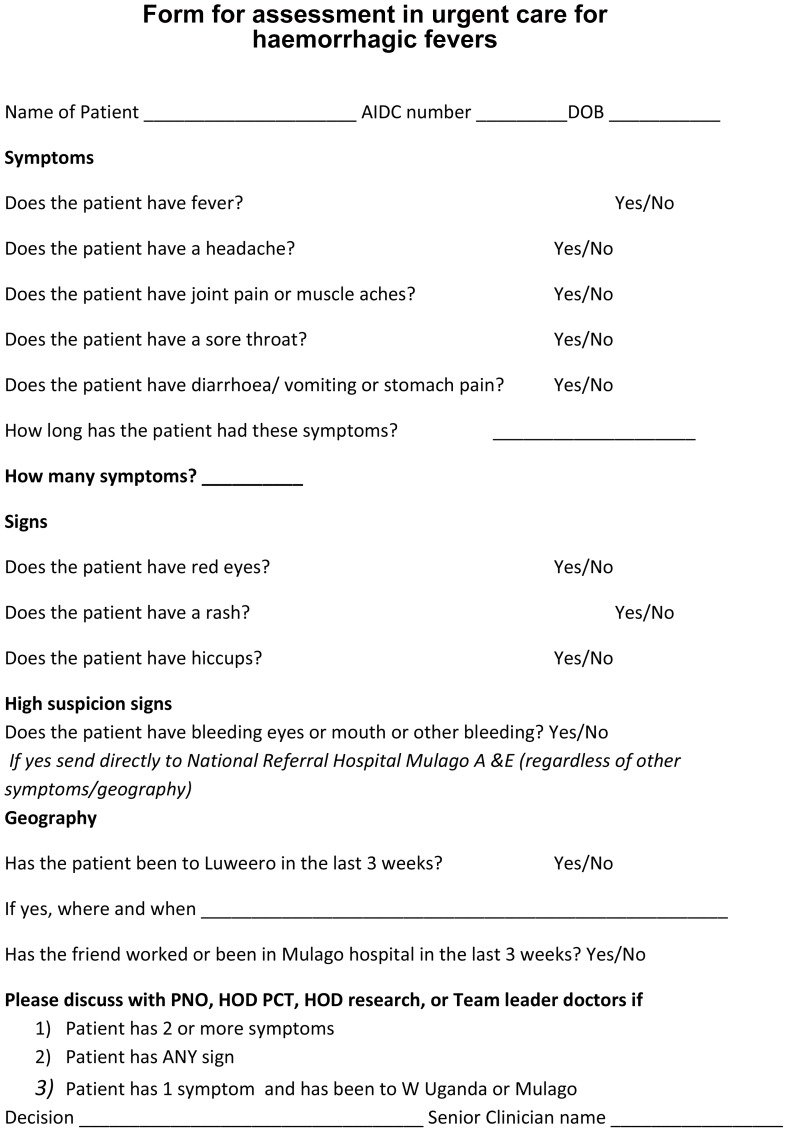
Form for assessment in urgent care for haemorrhagic fevers.

Symptomatic patients are seen and managed in a designated ‘urgent care’ area of the clinic. This is an isolated area, where patients can be seen and can wait apart from other patients. During the VHF outbreaks the staff working in this area were provided with additional protective clothing. All clinic doctors were provided with screening tool (figure 3) for use for all patients who had attended for an unscheduled visit, and any patients with scheduled appointments who reported symptoms suggestive of VHF not identified by the infection control nurse. The tool was reproduced and made available in all clinical rooms. The tool was mainly based on symptoms, and included a points system, where 2 or more symptoms or any sign, or one symptom in a patient having been to an outbreak area highlighted the patient as a possible VHF suspect. These were then discussed with a senior doctor for advice on referral to the VHF unit at the National Hospital. Cardinal signs of haemorrhagic necessitated an immediate referral to the National Hospital. A senior doctor visits the urgent care team frequently to provide support and advice, and this support was increased during the outbreaks. For this study we have reviewed the screening tools filled and the urgent care admissions list to find those considered to be possible VHF suspects during the study period.

**Figure 4:**
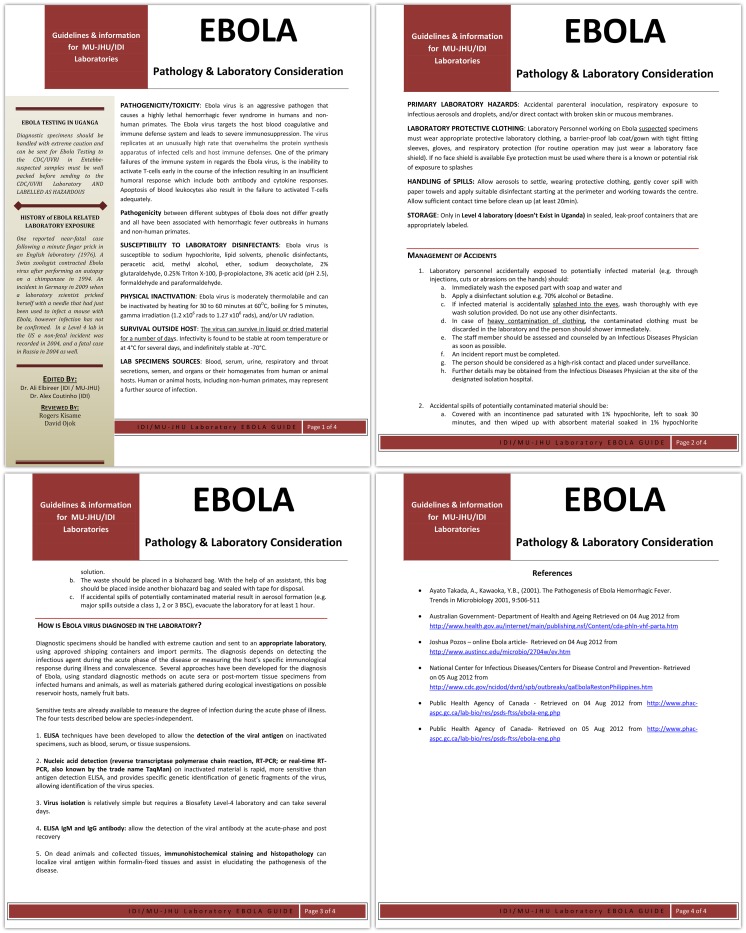
Laboratory Information –Ebola.

The internationally accredited IDI Core laboratory processes an average of 600 samples a day from over 40 sites around the country. The third tool was an information leaflet distributed to all lab staff and to all staff who were taking and dealing with blood and other samples (Figure 4).

Staff taking blood samples and working in the lab received sensitization on how to take samples, to manage samples and to dispose of samples in a safe manner.

### Ethical considerations

This process was approved by the Institutional Review Board. The study and use of data was reviewed and approved by the Scientific Review Board of the Infectious Diseases Institute, the Institutional Review Board of Makerere University and the Uganda National Council of Science and Technology. Individual written patient consent was waived by these ethical and review committees since data was collected for routine clinical care and unique identifiers are removed for operational research purposes.

## Results

### Outbreak 1 – Ebola Virus Disease Outbreak, Kibale (29th July 2012 to 4^th^ October 2012)

We developed the first draft of the tools within 24 hours of the WHO and Uganda Ministry of Health (MoH) announcements. The action plan was initiated and the screening tool was first distributed to staff on 30^th^ July 2012. The screening tool was then refined and re-circulated on 31^st^ July 2012. Over the 67 days of this outbreak, we saw 9458 patients in the clinic and of these 3077 were unscheduled appointments. The laboratory processed about 38,000 samples. The AIDC clinical team identified 2 possible suspects. Case 1 was female patient identified by the infection control nurse. She reported to the infection control nurse with a history of haematuria and diarrhea. She had not travelled to the VHF epicenter area and had not cared for patients from that area. The patient was seen in the urgent care isolation zone, with barrier nursing. Detailed further history revealed dysuria and diarrhea. She was afebrile and urine analysis was normal. She received ciprofloxacin and paracetamol and was discharged home. Case 2 was a female patient with fecal blood who had not been identified by the infection control nurse or the doctor, but reported bloody diarrhea to a counsellor, in the clinic. The counselor requested she see a doctor but the patient absconded from the clinic. She was contacted by telephone by a clinic doctor later that day and gave a 3 month history of altered bowel habit, with some per rectal bleeding, not consistent with an acute infection. This was worse after taking her ART. She had not travelled to the epicenter area and had not cared for patients from that area. VHF was ruled out on clinical history and was encouraged to come back for further investigations of her chronic problem. Case 3 had fever, headache, abdominal pain, and an itchy skin rash. She was afebrile, and she was treated for a non-specific rash with prurigo. She was treated with cetirizine, hydrocortisone cream and magnesium suspension for gastritis. She was discharged home that day.

### Outbreak 2 - Ebola Virus Disease (EVD) Outbreak, Luweero (17^th^ November and 30^th^ November 2012)

We instigated the process 24 hours before the WHO and MoH announcements (due to information received from Mulago Hospital) and an updated screening form were circulated to staff on 16^th^ November 2012 (figure 2 shows the response times in this outbreak). Over the 13 days of this outbreak we saw 3086 patients with HIV 1/2 infection in the clinic, and 636 had unscheduled appointments. The lab processed about 7,200 samples. We identified one possible suspect (case 4) who had a rash, fever, and diarrhea and was from Luweero district. This patient was given metronidazole and ciprofloxacin and transferred to the VHF unit at Mulago Hospital. Full infection control procedures were followed by staff handling the patient, and in handling laboratory samples. The patient was discharged from the VHF unit later in the day with no definite diagnosis. Laboratory samples were disposed of as per the protocol. Staff received counseling, but were not quarantined as they had no direct contact with body fluids. During the second outbreak we were contacted by another HIV service provider and research clinic working within the Mulago Hospital Complex, who requested our tools for use in their clinic.

### Outbreak 3 - Marburg Virus Disease Outbreak, South West Uganda (22^nd^ October-23^rd^ November 2012)

The process was not fully implemented as there were no reported suspects reaching Kampala, and the outbreak was mainly confined to the South West of Uganda.

### Post outbreak experiences (1^st^ December 2012–1^st^ July 2013)

During the 6 months following the outbreaks in Uganda, 10306 patients visited IDI (6032 unscheduled appointments). Clinic staff identified one VHF suspect (case 5) outside of a known outbreak in mid-2013. The patient presented with headache, fever, and diarrhea. He had epistaxis, hematemesis and bloody diarrhea. On examination he had hyperemic conjunctiva. He was found to have a thrombocytopenia with a platelet count of 114×10^3^/uL. This patient was admitted to the National Referral Hospital within 2 hours of being seen in the clinic. The patient was isolated on the ward, and hospital staff treated the patient as VHF suspect. Senior clinic staff were notified immediately. No staff member was exposed to patient fluids. Urgent communication to lab staff ensured that blood samples taken by staff were handled safely. The MOH national VHF response team was notified and he was tested by the MOH team for VHF within 24 hours of admission. The case was VHF serology negative. No definite diagnosis was found.

(Table 1 summarizes clinical information on all suspects seen)

**Table 1:** Summary of VHF suspect characteristics.

Patient	Age and Sex	Cd4 count	ART regimen	Symptoms	Possible exposure	Outcome
Case 1	46, F	592 (21%)	TDF/3TC/LPR-RIT	Dysuria, haematuria, diarrhea. No fever	Nil known	Treated for bacterial gastro-enteritis/UTI as outpatient with Ciprofloxacin
Case 2	42, F	196 (8%)	AZT/3TC/Nevirapine	Abdominal pain and diarrhoea after taking drugs	Nil known	Drug side effect – monitored as out patient
Case 3	35, F	492 (23%)	AZT/3Tc/Nevirapine	Headache, Abdominal pain, Rash	Nil known	PPE and gastritis – treated with cetirizine, hydrocortisone and magnesium suspension
Case 4	42, M	139 (13%)	TDF/3TC/EFV	Fever, joint pain, sore throat, diarrhoea	From district containing epicentre (Luweero)	Given treatment for bacterial gastroenteritis with ciprofloxacin/metronidazole. Transferred to National Referral Hospital VHF unit for investigation but discharged
Case -5	35,M	237 (17%)	Nil	Fever, headache, bloody diarrhoea, epistaxis, heamatemasis	Outside of known outbreak	Transferred to National Referral Hospital VHF unit

## Discussion

The VHF outbreaks in Uganda have caused a considerable amount of anxiety for both patients and staff in our large HIV clinic. In order to manage this anxiety and reduce the risk of occupational exposure in our clinic while providing an uninterrupted service we have designed and implemented easy to use tools in our clinic for use in VHF outbreaks. The action plan enabled us to communicate with staff within the clinic and in outreach projects around the country, as quickly and early as possible, in order to provide valuable information and to reduce anxiety. In both outbreaks we managed to communicate important information to staff within 48 hours of the announcements.

The use of an objective point based screened tool enabled us to continue to see a high number of HIV patients, often with vague symptoms, in a safe and effective manner in an out-patient HIV clinic which was not a VHF receiving centre. Our main objective was to provide a sensitive tool for identifying possible VHF suspects within a population of patients with HIV in order to refer them to a designated VHF clinic. Therefore, our tool was purposefully less specific than the WHO screening tools to be used in VHF centres, for instance, whilst we asked about symptoms, the questions we asked about exposure were limited to being in the geographical area, and did not go into details about contact with patients/attendance at burials etc. This did increase the number of “possible” suspects that were highlighted to a senior doctor, but our experience was that this was manageable number and that it was important to have a more sensitive tool that could be a primary screening, prior to more intensive screening by the VHF receiving centre.

The lessons learnt from the development of these tools included that having access to tools to follow also helped to reduce stress of the staff, as they were able to perform their normal duties with reassurance that not every patient they encountered might have VHF. During the second outbreak we were able to update the existing screening form very quickly and our response was more rapid and coordinated. Additionally the use of these tools has sensitized our staff on the symptoms and signs of VHF, and how to identify a possible suspect quickly. This was highlighted by the identification and rapid management of a possible suspect outside of a known outbreak. Whilst the IDI laboratory employs universal precautions at all times, management of suspected VHF samples requires a higher level of caution. The laboratory document enabled laboratory staff to understand what additional precautions they needed to take, and consequently to reduce anxiety by providing a procedure for management of samples.

Whilst our tool is “ideal” for our clinical environment, it does have to be adapted to each outbreak. What is “ideal” for us may not work in other clinics with different models of care e.g. nurse led services, services without an “urgent care” area. Perhaps the WHO or local Ministries of Health in countries at risk of outbreak could consider making these tools or adapted versions of these tools available for other out-patient clinic settings with high patient loads. Whilst our experience suggests that the action plan and the screening tools are easily understandable to staff, we did find that with multiple experiences we became quicker at implementing our response. Therefore, we think that health facilities may consider simple training programme or staff sensitization prior to an outbreak, which might also help health care workers to become familiar with the tools and to implement them quickly if an outbreak occurs.

## Conclusions

We believe that these tools/response aids may serve an unmet need in countries which suffer relatively frequent VHF outbreaks and have a high HIV prevalence. We would encourage other HIV clinics or clinics with a high turnover of patients, who work in areas of potential VHF outbreaks to consider a risk management strategy, with readily available and objective assessment tools, which can be activated quickly during a similar emergence outbreak situation.
